# Increased Circulating Soluble Junctional Adhesion Molecules in Systemic Sclerosis: Association with Peripheral Microvascular Impairment

**DOI:** 10.3390/life12111790

**Published:** 2022-11-04

**Authors:** Eloisa Romano, Irene Rosa, Bianca Saveria Fioretto, Marco Matucci-Cerinic, Mirko Manetti

**Affiliations:** 1 Section of Internal Medicine, Department of Experimental and Clinical Medicine, University of Florence, 50134 Florence, Italy; 2 Section of Anatomy and Histology, Department of Experimental and Clinical Medicine, University of Florence, 50134 Florence, Italy; 3 Unit of Immunology, Rheumatology, Allergy and Rare Diseases (UnIRAR), IRCCS San Raffaele Hospital, 20132 Milan, Italy

**Keywords:** systemic sclerosis, scleroderma, junctional adhesion molecules, sJAM-A, sJAM-C, peripheral microvascular damage, digital ulcers, enzyme-linked immunosorbent assay

## Abstract

Systemic sclerosis (SSc, scleroderma) is a severe disease characterized by peripheral microcirculation abnormalities manifesting with Raynaud’s phenomenon, nailfold videocapillaroscopic (NVC) changes, and even ischemic digital ulcers (DUs) that are often refractory to treatments. In the wake of previously described associations between the circulating levels of soluble junctional adhesion molecules (sJAMs) and SSc clinical features, here, we measured sJAM-A and sJAM-C levels by enzyme-linked immunosorbent assay in serum samples from a large case series of 110 SSc patients and 85 healthy controls, focusing on their possible association with peripheral vascular clinical features and their potential as biomarkers that are either diagnostic or mirror SSc-related microvasculopathy severity. Our data demonstrated that serum sJAM-A and sJAM-C are significantly increased in patients with SSc vs. healthy controls, especially in those featuring early/active NVC patterns and the presence of ischemic DUs. Moreover, circulating sJAM-C levels showed good diagnostic accuracy in discriminating between patients and controls, as assessed by receiver operator characteristics curve analysis. Finally, logistic regression revealed that, when comparing sJAM-A to sJAM-C, the latter might be better suited as a biomarker for SSc-related DUs. Our promising findings provide the necessary groundwork for longitudinal follow-up analyses of SSc patients aiming to assess whether circulating sJAM-C levels might be predictive for the development of new DUs, as well as DU recurrence and/or refractoriness to targeted therapies.

## 1. Introduction

Systemic sclerosis (SSc, scleroderma) is a severe disease affecting the connective tissue that is characterized by the combination of immunological disturbances, widespread peripheral microvasculopathy, and progressive cutaneous and visceral fibrosis [1,2,3]. In the early phases of the disease course, SSc patients experience peripheral circulatory disturbances that precede fibrosis by months or years and include microvascular tone dysregulation, clinically manifesting with Raynaud’s phenomenon and microangiopathy, as mirrored by nailfold capillaroscopic changes comprising capillary enlargement, bleeding, stenosis, and destruction [1,2,3]. Such microvascular dysfunction, characterized by endothelial cell (EC) activation/damage and perivascular infiltration/accumulation of lymphocytes and monocytes in the affected tissues [4,5], may frequently lead to significant peripheral ischemic manifestations such as digital ulcers (DUs) [5,6], i.e., disabling and painful lesions often resulting in infections and gangrene, heavily compromising patients’ quality of life [5,6]. Perivascular inflammation, which represents a typical hallmark of the early stages of the disease, is a consequence of an extravasation process mediated by the interaction between leukocytes and ECs, a mechanism highly reliant on the expression and function of cell surface adhesion molecules [7,8]. In addition to mediating leukocyte trafficking and inflammation, adhesion molecules play important roles in angiogenesis [9]. Indeed, endothelial adhesion molecules have been reported to regulate EC motility and to promote the influx of monocytes into tissues, where they can differentiate into macrophages and secrete proangiogenic factors, and to induce neovascularization with their soluble forms [9,10,11,12]. In this context, a number of studies suggested that, in the early stages of SSc, increased levels of EC-released soluble adhesion molecules may reflect the ongoing EC activation state and correlate with the presence and severity of specific organ complications [7,13,14].

Among the pool of cell-to-cell adhesion molecules, junctional adhesion molecule (JAM)-A (also known as JAM-1/F11 receptor) and JAM-C (JAM-3) are proteins belonging to the immunoglobulin superfamily and are broadly expressed on ECs, epithelial cells, fibroblasts, and circulating cells and are able to regulate leukocyte transmigration across the endothelium thanks to their ability to undergo heterophilic binding with the leukocyte integrins [15,16,17]. In addition, they can establish homophilic interactions at both endothelial and epithelial tight junctions, thus, participating in the regulation of paracellular permeability [15,16,17]. Since JAMs may also modulate cell adhesion, migration, and neovascularization, they have been implicated in several pathologic conditions, including cancer, hypertension, rheumatoid arthritis, and inflammatory bowel disease [18,19,20,21]. The soluble forms of these adhesion molecules have also been quantified in the circulation of patients affected by different pathologies, with their levels often correlating with disease severity [19,21,22,23,24,25,26]. As far as SSc is concerned, an abnormal expression of both JAM-A and JAM-C has been reported in the skin and blood of patients [7,27,28]. In particular, a previous study from our group demonstrated an aberrant expression of these JAMs not only in the skin of SSc patients, but also in SSc cultured dermal microvascular ECs, as well as in healthy microvascular ECs challenged with SSc sera [7]. Moreover, circulating levels of soluble JAM-A (sJAM-A) and sJAM-C were found to be significantly increased in patients with early-stage SSc and to correlate with different parameters of microvascular damage [7]. On the basis of such a preliminary scientific background and considering that our previous study was carried out on a relatively small cohort of patients, the aim of the present research was to evaluate sJAM-A and sJAM-C serum levels by enzyme-linked immune-sorbent assay (ELISA) in a larger case series of SSc patients, focusing on their association with peripheral vascular disease features and their possible potential as biomarkers that are either diagnostic or mirror SSc-related microvasculopathy severity.

## 2. Materials and Methods

### 2.1. Patients, Controls, and Serum Samples

Serum samples were obtained from 110 patients (101 women and 9 men; mean ± SD age 57.1 ± 14.2 years) recruited from the Division of Rheumatology and Scleroderma Unit, Azienda Ospedaliero-Universitaria Careggi (AOUC), Florence, Italy, and fulfilling the American College of Rheumatology/European League Against Rheumatism 2013 classification criteria for SSc [29]. SSc patients with symptoms which overlap with those of other autoimmune, rheumatic, and/or connective tissue diseases were excluded from the study. Patients were under vasoactive/vasodilating drugs as follows: calcium channel blockers (72.6%), endothelin receptor antagonists (17.6%), phosphodiesterase 5 inhibitors (16.5%), and iloprost (22.0%). Before blood sampling, they were washed out for 10 days from oral vasoactive/vasodilating drugs and for 2 months from intravenous iloprost. At the time of blood sample collection, patients were not under immunosuppressants or other disease-modifying drugs. Eighty-five age- and sex-matched healthy individuals (78 women, 7 men; mean ± SD age 58.5 ± 13.2 years) were used as controls. The presence of primary Raynaud’s phenomenon was considered as an exclusion criterion. Fresh venous blood samples from both patients and controls were drawn, allowed to clot for 30 min, and centrifugated at 1500× *g* for 15 min. Serum was then collected and stored in aliquots at −80 °C until used. The study was conducted in agreement with the Declaration of Helsinki and approved by the local institutional review board at the AOUC, Florence, Italy (approval number: AOUC 69/13; approval date: 17 June 2013). All the involved individuals provided written informed consent.

### 2.2. Clinical Assessment

Patients were classified as having limited cutaneous SSc (lcSSc; *n* = 72) or diffuse cutaneous SSc (dcSSc, *n* = 38) according to the criteria of LeRoy et al. [30] and phenotypically assessed as recommended [31]. All patients reported the presence of Raynaud’s phenomenon. At the time blood was drawn, the occurrence of ischemic DUs on the fingertips and other finger areas of SSc patients was recorded, and microvascular abnormalities on all 10 fingers were assessed by nailfold videocapillaroscopy (NVC). Briefly, patients were allowed to adapt to room temperature for at least 15 min and then their nailfolds were evaluated for the presence of pericapillary edema, microhemorrhages, enlarged and giant capillaries, ramified or bushy capillaries, disorganization of the vascular distribution, and loss of capillaries. The three different NVC patterns were identified as follows: (i) “early” NVC pattern, featuring few enlarged/giant capillaries and capillary microhemorrhages, no evident loss of capillaries and a relatively well-preserved capillary bed; (ii) “active” NVC pattern, characterized by giant capillaries and capillary microhemorrhages, absence/presence of few ramified capillaries, moderate capillary loss, and mild disorganization of the capillary structure; and (iii) “late” NVC pattern, with irregular capillary enlargement, absence/presence of few giant capillaries, no microhemorrhages, frequent ramified/bushy capillaries, severe loss of capillaries with large avascular areas, and disorganization of the normal capillary architecture [32]. A summary of the main features of the SSc patients is shown in Table 1.

### 2.3. Assay for Serum sJAM-A 

Serum levels of sJAM-A were quantified by commercial quantitative colorimetric sandwich ELISA using a 96-well microtiter plate precoated with a capture antibody specific to sJAM-A and a biotin-conjugated detection antibody (catalog no. EKU05421; Biomatik, Wilmington, DE, USA). Each sample was measured in duplicate. According to the manufacturer’s protocol, once both standards and serum samples (100 µL/well) were added to the wells, the plate was left to incubate for 1 h at 37 °C. The liquid was then removed without washing, and the wells were incubated for 1 h at 37 °C with 100 µL of detection reagent A (biotin-conjugated detection antibody). The reaction, developed by adding 100 µL/well of detection reagent B (HRP conjugate) and 90 µL/well of tetramethylbenzidine (TMB) substrate solution for 10–20 min at 37 °C, was stopped by the addition of 50 µL of a sulfuric acid stop solution. The optical density (OD) of each well was measured using a microplate reader at 450 nm, and the concentration of serum sJAM-A was determined by comparing the OD of the samples to the standard curve. The detection range and the sensitivity of the assay were 0.313–20 ng/mL and 0.127 ng/mL, respectively. 

### 2.4. Assay for Serum sJAM-C 

Serum levels of sJAM-C were measured by commercial ELISA (catalog no. E2769Hu; BT Lab, Shanghai, China), following the manufacturer’s instructions. Briefly, after adding standards (50 µL/well) and serum samples (40 µL/well) to the 96-well microplate precoated with an antibody specific to sJAM-C, 10 µL/well of a biotin-conjugated detection antibody and 50 µL/well of streptavidin–HRP were applied and left to incubate for 1 h at 37 °C. Next, following 5 washes, the reaction was developed by incubating the wells for 10 min at 37 °C in the dark with a 50 µL/well of substrate solution A and 50 µL/well of substrate solution B and, finally, terminated with 50 µL/well of stop solution. Color change was measured spectrophotometrically at a wavelength of 450 nm. The concentration of sJAM-C in the samples was determined by comparing the OD of each sample to those of the standard curve. The detection range and the sensitivity of the assay were 0.05–20 ng/mL and 0.021 ng/mL, respectively. Each sample was measured in duplicate. 

### 2.5. Statistical Analysis

Statistical data analysis was performed by means of the SPSS software for Windows Version 28.0 (SPSS, Chicago, IL, USA). Descriptive statistics for continuous variables were expressed as the mean ± SD or median and interquartile range (IQR), while those for categorical variables were reported as number and percentage. The non-parametric Mann–Whitney U test was used to assess serum sJAM-A or sJAM-C differences between two independent groups, while the Spearman ρ correlation coefficient was calculated to examine the relationship between two continuous variables. To verify the accuracy of circulating sJAM-A and sJAM-C levels for the diagnosis of SSc, the sensitivity (capability of the test to identify true positive subjects) and specificity (capability of the test to identify true negative subjects) of the test were evaluated for each molecule through receiver operator characteristics (ROC) curve analysis and the following estimation of the area under the curve (AUC) [33]. AUC values were interpreted as follows: 0.5–0.6 (failed), 0.6–0.7 (worthless), 0.7–0.8 (poor), 0.8–0.9 (good), >0.9 (excellent) [33]. Youden’s index (i.e., Sensitivity − (1 − Specificity)) was also applied in order evaluate the best cutoff value in our experimental data distributions. Since the Mann–Whitney U test unveiled that serum levels of both sJAMs were significantly increased in SSc patients with DUs and early/active NVC patterns, we performed multiple logistic regression analysis including sJAM-A and sJAM-C as independent variables and a single dependent variable each time (i.e., presence of DUs and NVC pattern). Odds ratios (ORs) with 95% confidence intervals (95% CIs) were determined. All *p*-values were two-tailed, and *p*-values < 0.05 were considered statistically significant. 

## 3. Results

### 3.1. Serum Levels of Both sJAM-A and sJAM-C Are Increased in SSc Patients

**Figure 1:**
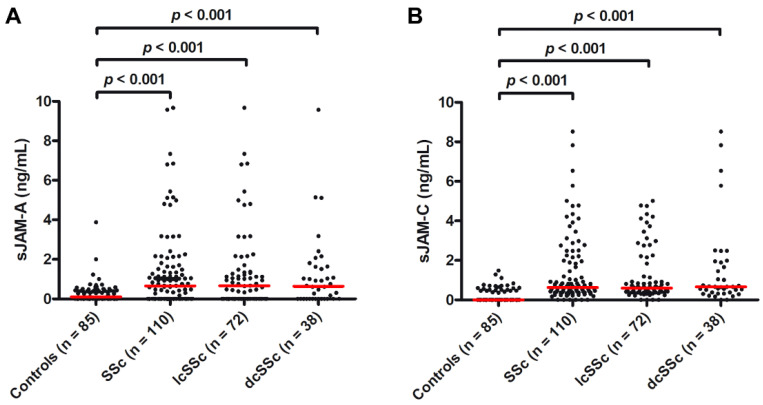
Serum levels of both (**A**) sJAM-A and (**B**) sJAM-C are increased in patients with SSc. (**A**) sJAM-A and (**B**) sJAM-C levels in healthy controls, patients with SSc, lcSSc, and dcSSc. Data are shown as dot plots. Each dot represents a subject. Horizontal red lines indicate the median value of each group. The non-parametric Mann–Whitney U test for independent samples was used to analyze serum sJAM-A and sJAM-C differences between groups. dcSSc, diffuse cutaneous SSc; lcSSc, limited cutaneous SSc; sJAM, soluble junctional adhesion molecule; SSc, systemic sclerosis.

Circulating levels of sJAM-A were significantly augmented in SSc patients (median 0.65 ng/mL, IQR 0.00–1.48 ng/mL) compared with healthy controls (median 0.20 ng/mL, IQR 0.00–0.42 ng/mL; *p* < 0.001; Figure 1A). sJAM-A levels were higher both in patients with lcSSc (median 0.66 ng/mL, IQR 0.00–1.36 ng/mL) and in those with dcSSc (median 0.64 ng/mL, IQR 0.00–1.63 ng/mL) than in controls (both *p* < 0.001; Figure 1A). Similarly, serum levels of sJAM-C were significantly increased in SSc patients (median 0.63 ng/mL, IQR 0.37–1.86 ng/mL) compared with healthy controls (median 0.00 ng/mL, IQR 0.00–0.46 ng/mL; *p* < 0.001; Figure 1B), as well as in both SSc cutaneous subsets vs. controls (lcSSc: median 0.60 ng/mL, IQR 0.37–1.68 ng/mL; dcSSc: median 0.66 ng/mL, IQR 0.37–1.91 ng/mL; both *p* < 0.001; Figure 1B). No significant difference in sJAM-A and sJAM-C levels was found between lcSSc and dcSSc patients. 

### 3.2. Diagnostic Accuracy of sJAM-A and sJAM-C for SSc

**Figure 2:**
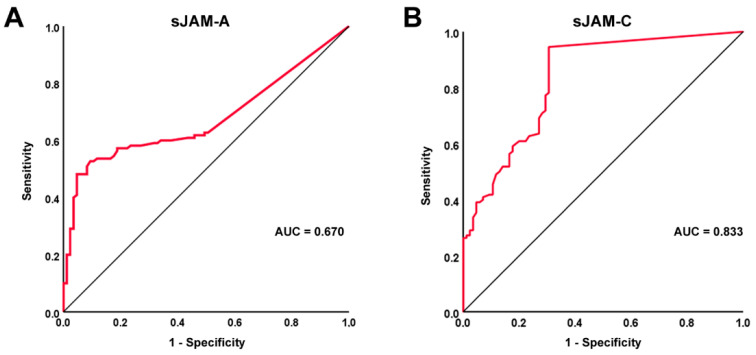
ROC curve (red line) plots for (**A**) sJAM-A and (**B**) sJAM-C in SSc patients vs. healthy controls. AUC values and reference lines for each curve are shown. AUC, area under the curve; ROC, receiver operator characteristic; sJAM, soluble junctional adhesion molecule; SSc, systemic sclerosis.

The ROC curves and the corresponding AUC were plotted in order to evaluate the diagnostic accuracy of the two assessed circulating molecules. In particular, the diagnostic accuracy of sJAM-A was found to be poor (AUC = 0.670, 95% CI 0.604–0.754), while the diagnostic accuracy of sJAM-C was found to be good (AUC = 0.833, 95% CI 0.775–0.891) (Figure 2A,B). In addition, for sJAM-C, the ROC curve analysis revealed a cutoff value of 0.09 ng/mL, with 94.5% sensitivity and 69.4% specificity in discriminating between SSc patients and healthy controls.

### 3.3. Association of sJAM-A and sJAM-C Serum Levels with the Severity of Microvascular Impairment

**Figure 3:**
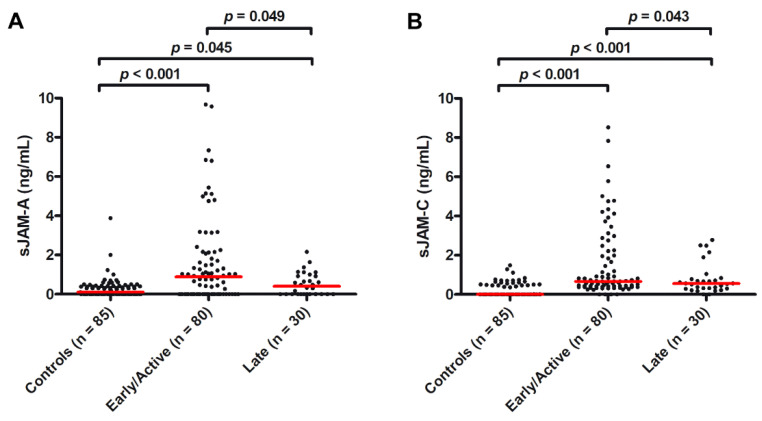
Serum levels of (**A**) sJAM-A and (**B**) sJAM-C in healthy controls and SSc patients stratified according to early/active and late nailfold videocapillaroscopic patterns. Data are shown as dot plots. Each dot represents a subject. Horizontal red lines indicate the median value of each group. The non-parametric Mann–Whitney U test for independent samples was used to analyze serum sJAM-A and sJAM-C differences between groups. sJAM, soluble junctional adhesion molecule; SSc, systemic sclerosis.

As a measure of peripheral microvascular involvement, we further investigated the presence of a possible association of serum sJAM-A and sJAM-C with the NVC pattern and the occurrence of ischemic DUs. As far as NVC is concerned, when compared to controls, both circulating levels of sJAM-A and sJAM-C were found to be significantly higher in SSc patients with either early/active NVC patterns (median 0.89 ng/mL, IQR 0.00–2.12 ng/mL for sJAM-A and median 0.66 ng/mL, IQR 0.40–2.16 ng/mL for sJAM-C; *p* < 0.001 for both molecules; Figure 3A,B) or a late NVC pattern (median 2.23 ng/mL, IQR 0.60–3.44 ng/mL for sJAM-A and median 0.66 ng/mL, IQR 0.40–2.16 ng/mL for sJAM-C; *p* = 0.045 for sJAM-A and *p* < 0.001 for sJAM-C; Figure 3A,B). When comparing sJAM levels between the two NVC groups, the values of both sJAM-A and sJAM-C were higher in the early/active pattern group compared to in the late one (*p* = 0.049 and *p* = 0.043, respectively; Figure 3A,B).

**Figure 4:**
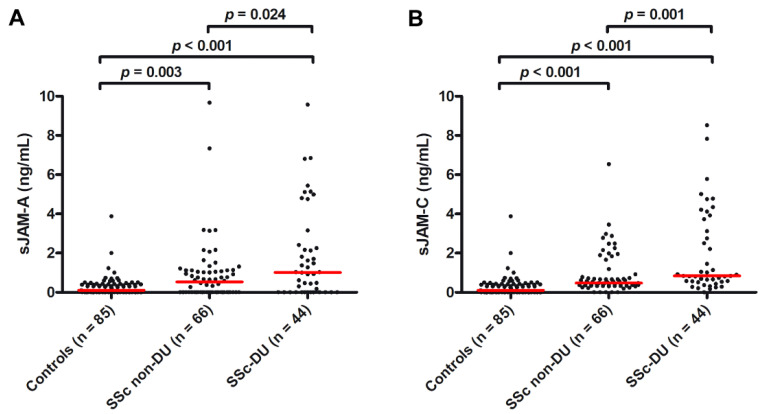
Serum levels of (**A**) sJAM-A and (**B**) sJAM-C in healthy controls and SSc patients with and without ischemic DUs. Data are represented as dot plots. Each dot represents a subject. Horizontal red lines indicate the median value of each group. The non-parametric Mann–Whitney U test for independent samples was used to analyze serum sJAM-A and sJAM-C differences between groups. DU, digital ulcers; sJAM, soluble junctional adhesion molecule; SSc, systemic sclerosis.

As far as ischemic DUs are concerned, the circulating levels of both sJAM-A and sJAM-C were found to be significantly higher when compared to controls both in SSc patients with DUs (median 1.01 ng/mL, IQR 0.00–2.37 ng/mL for sJAM-A and median 0.84 ng/mL, IQR 0.54–3.58 ng/mL for sJAM-C; *p* < 0.001 for both molecules; Figure 4A,B) and in those without DUs (median 0.53 ng/mL, IQR 0.00–1.12 ng/mL for sJAM-A and median 0.47 ng/mL, IQR 0.34–0.82 ng/mL for sJAM-C; *p* = 0.003 for sJAM-A and *p* < 0.001 for sJAM-C; Figure 4A,B). In addition, when comparing sJAM levels between the two SSc groups, the values of both sJAM-A and sJAM-C were significantly higher in patients with DUs compared to those without DUs (*p* = 0.024 and *p* = 0.001, respectively; Figure 4A,B).

### 3.4. Correlation between Serum Levels of sJAM-A and sJAM-C and Logistic Regression Model 

**Figure 5:**
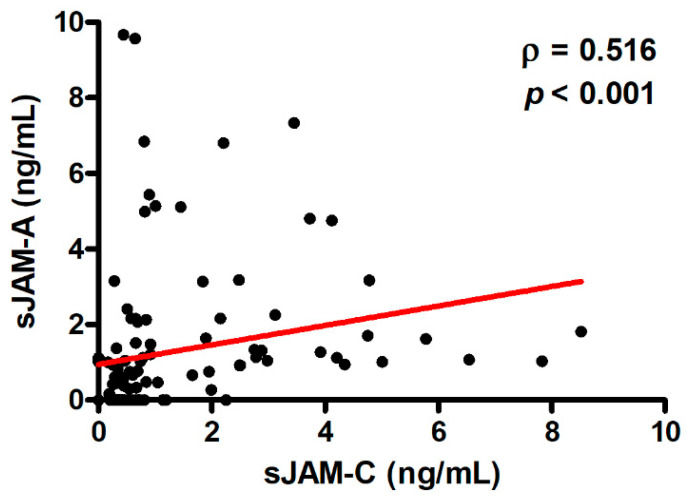
Correlation between sJAM-A and sJAM-C levels in patients with SSc. Data are displayed as a scatterplot where each dot represents a patient. Correlation coefficient (*ρ*) and *p*-values are indicated. sJAM, soluble junctional adhesion molecule; SSc, systemic sclerosis.

Circulating levels of sJAM-A and sJAM-C were directly correlated with one another in SSc patients (*ρ* = 0.516, *p* < 0.001; Figure 5) but not in healthy controls. 

Since, when comparing SSc subgroup medians, we found that serum levels of both sJAMs were significantly increased in SSc patients with DUs and early/active NVC patterns, we finally performed multiple logistic regression analysis combining serum sJAM-A and sJAM-C as independent variables and one of the two abovementioned disease phenotypes (i.e., presence of DUs and NVC pattern) as a single dependent variable each time. The results of the logistic regression analysis are shown in Table 2.

## 4. Discussion

In the wake of previously described associations between circulating levels of sJAMs and SSc [7,27], our data, obtained from a larger case–control cohort, allowed us to demonstrate that serum sJAM-A and sJAM-C were significantly increased in SSc vs. healthy controls, especially in patients characterized by early/active NVC patterns and the presence of ischemic DUs. Moreover, we showed for the first time that sJAM-C levels have a good diagnostic accuracy in discriminating between SSc patients and controls, as assessed by ROC curve analysis with cutoff value. Finally, logistic regression revealed that, when comparing sJAM-A to sJAM-C, the latter might be better suited as a biomarker for SSc-related DUs.

JAMs are cell adhesion molecules expressed by different cell types, including fibroblasts, in which they contribute to the establishment of their intercellular junctions and participate in intercellular crosstalk, retainment of myeloid cells, and wound healing [7,34,35], and ECs, in which they control tight junction maintenance and mediate leukocyte extravasation [21,36,37,38,39]. Due to their ability to trigger intracellular signal cascades at intercellular contact sites, JAMs are involved in different pathophysiologic processes such as leukocyte recruitment to sites of inflammation and ischemia–reperfusion injury, atherogenesis, angiogenesis, and fibrosis [7,20,35,38,39,40,41,42]. As far as angiogenesis is concerned, JAM-A was found to be crucial for proper EC motility, directional movement, and focal contact formation and was shown to be involved in EC migration through integrin αvβ3 [43] and to mediate basic fibroblast growth-factor-induced angiogenesis [40,44], an effect that was withdrawn in JAM-A deficient mice [45] or after EC-specific JAM-A depletion [40,46]. JAM-C has also been reported to act as a proangiogenic molecule, as its soluble form was demonstrated to induce in vitro angiogenesis [21], and its blockade was able to reduce tumor growth and decrease angiogenesis both in vivo and in vitro [47]. In line with these findings, overexpression of both JAM-A and JAM-C was shown to enhance tumor angiogenesis and metastasization in different in vitro and in vivo studies [48,49,50,51]. As already mentioned above, JAM-A and JAM-C are also supposed to be implicated in fibrosis. Indeed, JAM-A overexpression in mouse fibroblasts was demonstrated to significantly augment the secretory capacity and proliferation of these cells [35], while increased JAM-C expression was detected in experimental hepatic fibrosis [20,42,52]. 

In SSc, a disease in which an early proinflammatory state and microvasculopathy progressively culminate in fibrosis and loss of angiogenesis [53,54,55], aberrant JAM-A and JAM-C protein expression was reported in skin biopsies as well as in dermal fibroblasts and ECs [7,27,28]. Moreover, in a previous preliminary study from our group, circulating sJAM-A and sJAM-C levels were shown to be particularly high in patients with early-stage SSc compared to healthy controls, although no difference was found between the totality of patients and controls in the relatively small cohort analyzed [7]. At variance with our previous report, here, we demonstrate, in a much larger case–control cohort, that circulating sJAM levels are significantly increased in the whole group of SSc patients respective to the healthy subjects. Such a discrepancy may be explained either because our earlier study was underpowered or by the fact that, since here we aimed to assess the diagnostic potential of these molecules, we did not include SSc patients with longstanding disease. As demonstrated by the ROC curve analysis, we additionally revealed that sJAM-C levels might be a useful diagnostic tool for discriminating between SSc and a healthy condition, evidence that is further supported by the fact that sJAM-C in patients with SSc was always significantly higher than in controls regardless of the clinical subgroup considered.

Interestingly, increased sJAM levels have been described in other different pathologic conditions. In particular, sJAM-A was found to be higher in patients with coronary artery disease, arterial hypertension, renal insufficiency, and multiple myeloma [19,23,24,26,56], while sJAM-C was shown to be augmented in rheumatoid arthritis and macular degeneration [21,25]. Consistent with our previously published results [7], we herein confirm in a larger sample that both sJAM-A and sJAM-C are predominantly higher in patients with early/active NVC patterns, which are characterized by an ongoing dysregulated angiogenic response resulting in microhemorrhages and immature and unstable giant microvessels, suggesting that an increase in sJAMs might not only reflect but possibly also actively contribute to the derangement of peripheral microcirculation in SSc patients. In addition, we not only validated the previously reported association between higher circulating levels of both sJAMs and SSc-related DUs, but, through logistic regression, also uncovered sJAM-C as a better biomarker for more severe peripheral vasculopathy characterized by the development of ischemic DUs. Strikingly, the potential of serum sJAM-C levels as a biomarker was also suggested for wet, age-related macular degeneration, which is characterized by vascular abnormalities into the macula [25].

The presence of soluble JAMs in the circulation depends on the cleavage of the extracellular domain of cell surface JAMs [21,57,58,59]. In particular, it has been reported that, under inflammatory conditions, the endothelial JAM-A and JAM-C extracellular domains are cleaved and secreted as soluble forms by a disintegrin and metalloproteinases (ADAMs) as well as neutrophil elastases [21,57,59]. Of note, patients with SSc show both an augmented protein expression of cell-membrane-bound JAM-A and JAM-C, and a significant upregulation of ADAM-17 and ADAM-12 proteins [7,60,61], which, together, might partly explain the increased concentrations of sJAM-A and sJAM-C in the circulation of SSc patients. To date, little is known about the mechanisms of action and/or the signaling cascades initiated by sJAMs. However, it has been hypothesized that sJAM-C may bind to membrane-bound JAM-B or JAM-C on the surface of ECs in order to initiate angiogenesis through the phosphorylation of Src, p38, and PI3K [21]. In addition, another study showed that the extracellular domain of JAM-C is cleaved and secreted in soluble form from adipose-derived stem cells, and released sJAM-C was demonstrated to couple with JAM-B to promote cell adhesion, cell growth, and the expression of mesenchymal markers in such cells [62]. On the basis of the aforementioned background, our findings allow us to speculate that sJAM-C might be implicated in both the vascular and fibrotic aspects of SSc.

Based on the present data, we are aware that further studies are necessary to determine if serum levels of both sJAM-A and sJAM-C may correlate with additional clinical SSc manifestations. Furthermore, longitudinal studies monitoring changes in levels of sJAM-C in patients with a very early diagnosis of SSc [63] who progress into an established SSc disease will help us to gain further insights into the potential diagnostic value of this circulating molecule. Prospective follow-up analyses of SSc patients are also required to fully unravel whether sJAM-C might be suited as a risk biomarker for the development of new DUs, as well as DU recurrence and/or refractoriness to treatments. We also believe that additional in vitro and in vivo studies will help us to further explore the role of JAMs in SSc pathophysiology and to unveil whether the modulation of their expression/activity will allow us to find new targeted therapeutic strategies for the treatment of this devastating pathology. Finally, we are confident that our promising results will stimulate further research aimed at evaluating circulating levels of these adhesion molecules in other pathologic conditions characterized by microcirculatory alterations and tissue fibrosis.

## Figures and Tables

**Table 1:** Demographic and clinical characteristics of SSc patients.

Characteristics	SSc Patients (*n* = 110)
Age, mean ± SD (years)	57.1 ± 14.2
Sex	
Male	9 (8.2)
Female	101 (91.8)
Disease subset	
lcSSc	72 (65.5)
dcSSc	38 (34.5)
Disease duration, mean ± SD (years)	4.3 ± 2.9
Digital ulcers	44 (40.0)
NVC pattern	
Early	36 (32.7)
Active	44 (40.0)
Late	30 (27.3)
Vasoactive/vasodilator drugs	
Calcium channel blockers	66 (60.0)
Endothelin receptor antagonists	16 (14.5)
Phosphodiesterase 5 inhibitors	15 (13.6)
Iloprost	20 (18.2)

Except where indicated otherwise, values are *n* (%) of subjects. dcSSc, diffuse cutaneous SSc; lcSSc, limited cutaneous SSc; NVC, nailfold videocapillaroscopy; SSc, systemic sclerosis.

**Table 2:** Logistic regression analysis model combining serum sJAM-A and sJAM-C levels in SSc patients.

		Early/Active NVC	DUs
sJAM-A	OR (95% CI)	0.65 (0.41–1.04)	1.28 (1.01–1.61)
	*p*	0.073	0.04
sJAM-C	OR (95% CI)	0.75 (0.50–1.15)	1.48 (1.12–1.96)
	*p*	0.189	0.007

CI, confidence interval; DUs, digital ulcers; NVC, nailfold videocapillaroscopy; OR, odds ratio; sJAM, soluble junctional adhesion molecule; SSc, systemic sclerosis.

## Data Availability

All relevant data are included within the manuscript.
